# Familial Moyamoya disease associated with dual RNF213 variants (R4810K and T1727M): A case report and genetic investigation

**DOI:** 10.1097/MD.0000000000047079

**Published:** 2026-01-23

**Authors:** Ben Sang, Wenpeng Lu, Lei Feng

**Affiliations:** a Department of Neurosurgery, Jining NO.1 People’s Hospital Affiliated to Shandong First Medical University, Jining, Shandong Province, China; b Jining Key Laboratory of Stroke and Neural Repair, Jining, Shandong Province, China; c Institute of Central Neurovascular Injury and Repair of Jining Medical Research Academy, Jining, Shandong Province, China.

**Keywords:** Moyamoya disease, point mutation, R4810K, RNF213, T1727M

## Abstract

**Background:**

Moyamoya disease (MMD) is a progressive cerebrovascular disorder characterized by stenosis or occlusion of the distal internal carotid arteries, frequently observed in East Asian populations. The RNF213 R4810K variant is a well-established susceptibility factor for MMD; however, the pathogenic significance of other rare variants, such as T1727M, remains largely undefined. The co-occurrence and potential synergistic effects of dual RNF213 variants have rarely been documented.

**Methods:**

We investigated a 3-generation family consisting of 17 members following the diagnosis of MMD in the proband. Comprehensive clinical evaluations, magnetic resonance angiography, and whole-exome sequencing were performed on symptomatic individuals, while targeted genotyping for RNF213 and other candidate variants was conducted on the remaining family members.

**Results:**

The proband was a 63-year-old woman with a history of ischemic and hemorrhagic cerebrovascular events. Genetic testing revealed 2 RNF213 variants, R4810K and T1727M, which were subsequently identified in 10 family members. Among these, 5 were diagnosed with MMD, 1 exhibited large-vessel occlusion with stroke symptoms, and 4 were asymptomatic but are currently under clinical surveillance. Additional rare variants were identified in HTRA1, PROS1, and F13A1, which may further modulate disease expression.

**Conclusion:**

We report for the first time a multigenerational case with dual RNF213 variants (R4810K and T1727M) and modifiers in familial MMD. The findings underscore the importance of genetic screening in at-risk families and suggest that dual RNF213 mutations may increase disease penetrance. Further mechanistic studies are warranted to elucidate gene–gene interactions and the role of additional modifiers in MMD pathogenesis.

## 1. Introduction

Moyamoya disease (MMD) is a rare cerebrovascular condition characterized by progressive stenosis and eventual occlusion of the distal internal carotid arteries, accompanied by the development of fragile collateral vessels that resemble a “puff of smoke” on angiography. Clinical manifestations vary and may include ischemic stroke, intracerebral hemorrhage, headache, and cognitive decline, among others.^[1]^ The RNF213 gene has been identified as the major susceptibility gene for MMD, particularly in East Asian populations.^[2,3]^ While the R4810K variant has been well established as a pathogenic mutation, the role of other RNF213 variants, Many mutations at other RNF213 sites have also been reported in the literature,^[4,5]^ such as T1727M, remains uncertain. Although several case reports have described MMD progression in patients harboring RNF213 polymorphisms.^[6]^ A case of a female patient with systemic lupus erythematosus who was diagnosed with a double mutation at the RNF213 site, the p.Arg4810Lys (R4810K) and the p.Thr1727Met (T1727M), supplemented the clinical symptoms that this mutation may cause.^[7]^familial cases involving both R4810K and T1727M variants in large pedigrees are extremely rare.

In this report, we present a multigenerational family with several members diagnosed with MMD, in which dual variants in RNF213 (R4810K and T1727M) were consistently identified.

## 2. Case report

**Figure 1.**
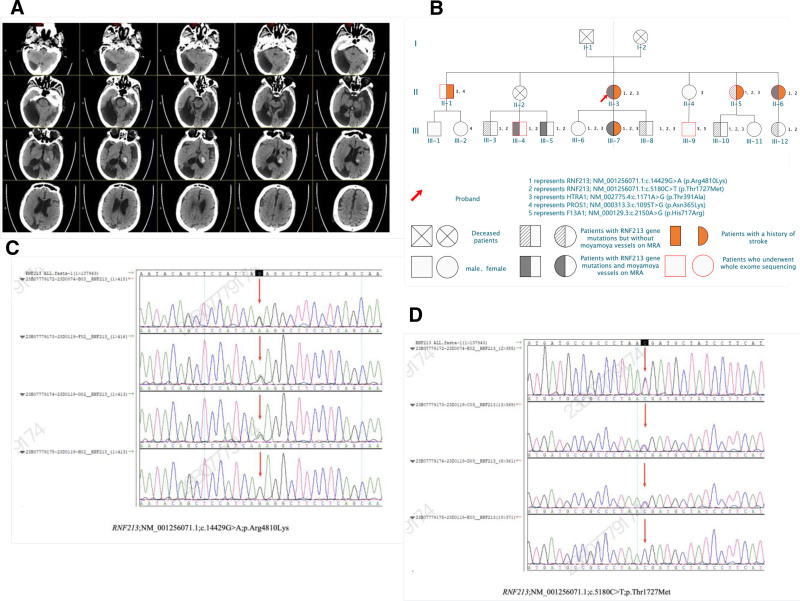
(A) Cranial CT showing a left basal ganglia hemorrhage in the proband. (B) Family pedigree. (C) Sequencing result of RNF213 (c.14429G > A, p.Arg4810Lys). (D) Sequencing result of RNF213 (c.5180C > T, p.Thr1727Met).

The proband of this family was a 63-year-old woman who presented with a left basal ganglia hemorrhage (Fig. 1A). She had a prior history of cerebral infarction in 2003. In 2016, she experienced transient limb weakness and dysarthria, and digital subtraction angiography revealed findings suggestive of Moyamoya disease. However, surgical intervention was declined at that time.

During her most recent hospitalization, a cranial CT scan confirmed a small-volume intracerebral hemorrhage (<10 mL) localized to the left basal ganglia. Her recorded peak systolic blood pressure was 150 mm Hg, and she reported no other chronic medical conditions.

The extended family includes 17 surviving members (Fig. 1B), prompting further evaluation for potential familial aggregation of disease.

## 3. Family history

A detailed family history was obtained from all 17 surviving members (Table 1; Fig. 1B), encompassing 3 generations. Key clinical information is summarized as follows:

**Table 1:** Family clinical timeline.

Generation/ Member	Age(year)	Previous clinical event(s)	Imaging/ Diagnosis	RNF213 mutations(Y/N)
I-1 (Father)	Died at 55	Esophageal cancer	Not checked	Not tested
I-2 (Mother)	Died at 67	Coronary disease; intracerebral hemorrhage	Not checked	Not tested
II-1 (Brother)	70	Myocardial infarction, cerebral infarction	Abnormal MRA	N
III-1 (Son)	46	Healthy	Normal MRA	N
III-2 (Daughter)	44	Dizziness	Normal MRA	N
II-2 (Sister)	Died at 68	Gallbladder disease	Not checked	Not tested
III-3 (Son)	42	Healthy	Normal MRA	Y
III-4 (Son)	38	Dizziness	MMD confirmed	Y
III-5 (Son)	35	Epilepsy	MMD confirmed	Y
II-3 (Proband)	63	Cerebral infarction;Cerebral hemorrhage	MMD confirmed	Y
III-6 (Daughter)	37	Healthy	Normal MRA	N
III-7 (Daughter)	36	Cerebral infarction	MMD confirmed	Y
III-8 (Son)	32	Healthy	Normal MRA	Y
II-4 (Sister)	56	Healthy	Normal MRA	N
III-9 (Son)	31	Dizziness	Normal MRA	N
II-5 (Sister)	55	Cerebral hemorrhage; cerebral infarction	Abnormal MRA	Y
III-10 (Son)	28	Healthy	Normal MRA	Y
III-11 (Daughter)	20	Healthy	Normal MRA	N
II-6 (Sister)	49	Cerebral hemorrhage	MMD confirmed	Y
III-12 (Daughter)	24	Healthy	Normal MRA	Y

### 3.1. First generation

- I-1 (Father): Deceased at age 55 due to esophageal cancer.
- I-2 (Mother): Had coronary artery disease and hypertension; died of intracerebral hemorrhage at age 67.

### 3.2. Second generation

- II-1 (Older brother): Male, 70 years old. History of myocardial infarction and cerebral infarction; underwent cardiac stent placement and left temporal lobe infarction. Current magnetic resonance angiography (MRA) shows signs of atherosclerosis.

1) III-1 (Son), 46 years old, healthy.
2) III-2 (Daughter), 44 years old, has occasional dizziness; cranial MRA is normal.
• II-2 (Older sister): Deceased at age 68 due to gallbladder disease.
1) III-3 (Son): 42 years old, healthy.
2) III-4 (Son): 38 years old, diagnosed with MMD at age 36 following dizziness; underwent left-sided bypass surgery.
3) III-5 (Son): 35 years old, diagnosed with MMD in the same year; has epilepsy.
• II-3: The proband (described above).
1) III-6 (Daughter): 37 years old, healthy.
2) III-7 (Daughter): 36 years old, MMD confirmed on MRA.
3) III-8 (Son): 32 years old, healthy; normal cranial MRA.

• II-4 (Younger sister): 56 years old, healthy; normal MRA.

1) III-9 (Son): 31 years old, occasional dizziness; MRA normal.

• II-5 (Younger sister): 55 years old, history of mild history of mild hemiparesis; MRA shows right P2 occlusion, right C3 bulge, and chronic hemorrhage in the right basal ganglia.

1) III-10 (Son): 28 years old, healthy; normal MRA.
2) III-11 (Daughter): 20 years old, healthy; normal MRA.

• II-6 (Youngest sister): 49 years old, diagnosed with MMD at age 39; underwent bilateral bypass surgeries and developed basal ganglia hemorrhage 6 months after the second surgery.

1) III-12 (Daughter): 24 years old, healthy; normal MRA.

## 4. Genetic testing

Based on the clustering of cerebrovascular events, we recommended genetic testing for the proband and her extended family. Among the 17 family members, 6 symptomatic individuals (the proband, her older brother, her second sister’s second and third sons, her fourth sister, and her fifth sister) underwent whole-exome sequencing.

Sequencing identified 5 variants of interest:

- RNF213; NM_001256071.1: c.14429G > A (p.Arg4810Lys)–Figure 1C
- RNF213; NM_001256071.1: c.5180C > T (p.Thr1727Met)–Figure 1D
- HTRA1; NM_002775.4: c.1171A > G (p.Thr391Ala)
- PROS1; NM_000313.3: c.1095T > G (p.Asn365Lys)
- F13A1; NM_000129.3: c.2150A > G (p.His717Arg)

The remaining 11 family members were specifically screened for these 5 mutations. Of the 17 total individuals, 10 were found to carry both the RNF213 R4810K and T1727M variants. Among them:

- 5 were definitively diagnosed with MMD via cranial MRA or digital subtraction angiography
- 1 (II-5) had large-vessel occlusion and stroke symptoms without formal MMD diagnosis
- 4 were asymptomatic (aged 42, 32, 28, and 24) and had normal MRA results

These asymptomatic carriers are being closely monitored through longitudinal follow-up to assess potential disease progression.

## 5. Discussion

This familial case provides novel insight into the genetic complexity of MMD, particularly regarding the coexistence of dual RNF213 variants and potential modifier genes. In East Asian populations, the p.Arg4810Lys (R4810K) variant is well established as a major susceptibility allele that significantly increases the risk of MMD.^[2,3]^ Conversely, the role of the p.Thr1727Met (T1727M) variant remains poorly characterized. The consistent co-segregation of both variants in this multigenerational pedigree, together with the observed penetrance of cerebrovascular disease (60% among carriers), suggests a potential cumulative or synergistic effect, as reflected by the notably high incidence of Moyamoya disease (approximately 50%) among individuals harboring both variants. This aligns with the “second-hit hypothesis,” whereby R4810K provides a predisposing background and additional variants, such as T1727M, may modulate age of onset, severity, or clinical phenotype.^[8,9]^

In 2003, a nationwide epidemiological survey of moyamoya disease in Japan, which has the highest incidence rate in Asia, showed that the incidence and prevalence rates were 6.03/100,000 and 0.57/100,000 respectively.^[10]^ In this multigenerational family of 17 members, it is significantly higher than the epidemiological data in Japan. In an international cohort study of the genetic map of childhood moyamoya disease, Paolo Zanoni et al found that among 88 children with moyamoya disease, there were 6 children carrying pathogenic RNF213 double-site variants. These 6 children may represent a subgroup with a more severe phenotype and a higher risk of extracranial vascular lesions, suggesting that gene dosage or compound variants may aggravate disease manifestations.^[11]^ A study by Ayako Kashimada et al found that mutations at the p.His4058Pro and p.Thr4155Pro sites in RNF213 can cause moyamoya disease and middle aortic syndrome, revealing its role in innate immune activation, complement cascade upregulation, and lung development disorders, breaking through the previous understanding that only focused on cerebrovascular diseases.^[12]^These observation raises the possibility of a synergistic or additive effect of the 2 variants, consistent with the “second-hit” hypothesis proposed in previous studies.^[9]^ While R4810K may act as a strong predisposing allele, the presence of T1727M may modulate the severity, age of onset, or phenotypic expression of MMD.

Beyond RNF213, our genetic analysis identified rare variants in HTRA1, PROS1, and F13A1, which may act as additional modifiers. HTRA1 encodes a serine protease implicated in vascular remodeling and smooth muscle integrity; its mutations are known to cause cerebral small vessel disease.^[13]^ PROS1 encodes protein S, a cofactor in anticoagulation pathways, where dysfunction may predispose to thrombosis or ischemic injury.^[14]^ F13A1 encodes coagulation factor XIII, crucial for fibrin crosslinking and vascular stability; its variants could enhance vascular fragility.^[15]^ Although individually of uncertain significance, these variants may collectively exacerbate endothelial dysfunction, inflammation, or hemostatic imbalance, thereby intensifying the pathogenic effect of RNF213 mutations. The phenotypic heterogeneity among carriers in our cohort supports the notion that MMD is a multifactorial disease, influenced not only by RNF213 mutations but also by modifier genes, epigenetic regulation, and environmental exposures.^[9,11]^

The phenotypic variability observed within this family underscores the multifactorial nature of MMD. Despite identical dual RNF213 variants, some individuals remained asymptomatic into adulthood, while others developed early or severe disease. This highlights the role of modifier genes, epigenetic regulation, and environmental influences (e.g., hypertension, autoimmune disease, infections) in shaping disease expression.

Mechanistically, RNF213 is a large E3 ubiquitin ligase and AAA + ATPase that plays roles in angiogenesis, lipid metabolism, and immune regulation.^[16]^ Experimental studies have shown that R4810K impairs angiogenic sprouting and endothelial migration.^[17]^ However, the functional effects of T1727M remain unknown. It is plausible that dual mutations affect different functional domains of RNF213, leading to compound dysfunction of vascular development pathways. In vitro studies using endothelial or smooth muscle cells expressing both mutations may help clarify this hypothesis.

From a clinical perspective, this report emphasizes the necessity of genetic screening in at-risk families. Asymptomatic carriers of dual RNF213 variants should be carefully monitored with serial neuroimaging, given their lifelong risk of ischemic or hemorrhagic events. Furthermore, identifying additional modifier variants may eventually inform risk stratification, prognostic assessment, and personalized surveillance strategies.

Taken together, the findings from this family highlight the potential cumulative pathogenic burden of coexisting RNF213 variants and provide a basis for future research into the genetic architecture and clinical management of familial Moyamoya Disease, and it provides a framework for future mechanistic and clinical investigations.

## Acknowledgments

This work was supported by Jining No.1 People’s Hospital, affiliated with Shandong First Medical University and the Shandong Academy of Medical Sciences. We also acknowledge the support of the Shandong Provincial Key Medical and Health Laboratory of Neuroinjury and Repair.

## Author contributions

**Conceptualization:** Lei Feng.

**Data curation:** Wenpeng Lu.

**Investigation:** Wenpeng Lu.

**Resources:** Lei Feng.

**Supervision:** Lei Feng.

**Writing – original draft:** Ben Sang.

**Writing – review & editing:** Ben Sang.
